# Supplementary material for: Decision-Making Preferences among Advanced Cancer Patients in a Palliative Setting in Jordan
Source: Int J Environ Res Public Health. 2023 Apr 18;20(8):5550. doi: 10.3390/ijerph20085550 (PMC10138437; doi:10.3390/ijerph20085550)
Supplement: Supplementary file 1 [file ijerph-20-05550-s001.zip › ijerph-2235861-supplementary.pdf]

Table S1. Control Preference Scale (Patient–Family–Physician items) as defined by Yennurajalingam et al. in 2017 [9].

In my opinion, decisions about my care should be made (please choose ONLY ONE):
1. By myself.
2. By myself after hearing the doctor's opinion or input.
3. By myself after hearing my family's opinion or input.
4. By myself after hearing both my family's and the doctor's opinion or input.
5. By my family.
6. By my family after hearing my opinion or input.
7. By my family after hearing my doctor's opinion or input.
8. By my family after hearing both my doctor's and my opinion or input.
9. By the doctor.
10. By the doctor after hearing my opinion or input.
11. By the doctor after hearing my family's opinion or input.
12. By the doctor after hearing both my family's and my opinion or input.
13. Shared between myself and the doctor.
14. Shared between myself and my family.
15. Shared between myself and both my family and doctor.

Table S2. Actual decision making scale (Patient–Family–Physician items) as defined by Yennurajalingam et al. in 2017 [9].

How were the decisions about your care actually made? (please choose ONLY ONE)
1. By myself.
2. By myself after hearing the doctor's opinion or input.
3. By myself after hearing my family's opinion or input.
4. By myself after hearing both my family's and the doctor's opinion or input.
5. By my family.
6. By my family after hearing my opinion or input.
7. By my family after hearing my doctor's opinion or input.
8. By my family after hearing both my doctor's and my opinion or input.
9. By the doctor.
10. By the doctor after hearing my opinion or input.
11. By the doctor after hearing my family's opinion or input.
12. By the doctor after hearing both my family's and my opinion or input.
13. Shared between myself and the doctor.
14. Shared between myself and my family.
15. Shared between myself and both my family and doctor.

Table S3. Control Preference Scale (Patient–Physician items) as defined by Yennurajalingam et al. in 2017 [9].

1. I would prefer to make the treatment decisions on my own.
2. I would prefer to make the treatment decisions by myself after hearing my doctor's opinion or input.
3. I would prefer to make the decisions together with my doctor.
4. I would prefer that the doctor make the decisions after talking to me and hearing my opinion.
5. I would prefer that my doctor make the decisions on his/her own.

Table S4. Control Preference Scale (Patient–Family items) as defined by Yennurajalingam et al. in 2017 [9].

1. I would prefer to make the treatment decisions on my own.
2. I would prefer to make the treatment decisions by myself after hearing my family's opinion or input.

3.	I would prefer to make the decisions together with my family.
4.	I would prefer that my family make the decisions after talking to me and hearing my opinion.
5.	I would prefer that my family make the decisions on his/her/their own.

Table S5. Patients' satisfaction with decision-making as as difined by Yennurajalingam et al. in 2017 [9].

Please Rate your Agreement with the Following Statements					
	**0 Completely disagree**	**1 Disagree**	**2 Undecided**	**3 Agree**	**4 Completely Agree**
a. I am satisfied with the information I received about my care					
b. I am satisfied with the way decisions about my care were made					
c. I am satisfied with the decisions about my care					

Table S6. Performance status as defined by Karnofsky, Burchenal, and their colleagues in 1948.

Definition	Performance Status %	Criteria
Able to carry on normal activity and to work. No special care is needed.	100%	Normal; no complaints; no evidence of disease.
	90%	Able to carry on normal activity; minor signs or symptoms of disease.
	80%	Normal activity with effort; some signs or symptoms of disease.
Unable to work. Able to live at home, care for most personal needs. A varying amount of assistance is needed.	70%	Cares for self. Unable to carry on normal activity or to do active work.
	60%	Requires occasional assistance, but is able to care for most of his needs.
	50%	Requires considerable assistance and frequent medical care.
Unable to care for self. Requires equivalent of institutional or hospital care. Disease may be progressing rapidly.	40%	Disabled; requires special care and assistance.
	30%	Severely disabled; hospitalization is indicated although death not imminent.
	20%	Very sick; hospitalization necessary; active supportive treatment necessary.
	10%	Moribund; fatal processes progressing rapidly.
	0%	Dead.
